# Modification of substrate specificity of l-arginine oxidase for detection of l-citrulline

**DOI:** 10.1186/s13568-023-01636-6

**Published:** 2023-12-03

**Authors:** Kei Yamamoto, Yosuke Masakari, Yasuko Araki, Atsushi Ichiyanagi, Kotaro Ito

**Affiliations:** 1 Marketing and Planning Division, Kikkoman Biochemifa Company, 1600, Kaisuka, Kamogawa, Chiba 296-0004 Japan; 2 Research and Development Division, Kikkoman Corporation, 338 Noda, Noda, Chiba 278-0037 Japan

**Keywords:** l-citrulline, l-arginine oxidase, l-citrulline dehydrogenase, Modification of substrate specificity

## Abstract

Enzymatic detection of citrulline, a potential biomarker for various diseases, is beneficial. However, determining citrulline levels requires expensive instrumental analyses and complicated colorimetric assays. Although l-amino acid oxidase/dehydrogenase is widely used to detect l-amino acids, an l-citrulline-specific oxidase/dehydrogenase has not been reported. Therefore, in this study, we aimed to develop an l-citrulline-specific enzyme by introducing a mutation into l-arginine oxidase (ArgOX) derived from *Pseudomonas sp.* TPU 7192 to provide a simple enzymatic l-citrulline detection system. The ratio of the oxidase activity against l-arginine to that against l-citrulline (Cit/Arg) was 1.2%, indicating that ArgOX could recognize l-citrulline as a substrate. In the dehydrogenase assay, the specific dehydrogenase activity towards l-arginine was considerably lower than the specific oxidase activity. However, the specific dehydrogenase activity towards l-citrulline was only slightly lower than the oxidase activity, resulting in improved substrate specificity with a Cit/Arg ratio of 49.5%. To enhance the substrate specificity of ArgOX, we performed site-directed mutagenesis using structure-based engineering. The 3D model structure indicated that E486 interacted with the l-arginine side chain. By introducing the E486 mutation, the specific dehydrogenase activity of ArgOX/E486Q for l-citrulline was 3.25 ± 0.50 U/mg, which was 3.8-fold higher than that of ArgOX. The Cit/Arg ratio of ArgOX/E486Q was 150%, which was higher than that of ArgOX. Using ArgOX/E486Q, linear relationships were observed within the range of 10–500 μM l-citrulline, demonstrating its suitability for detecting citrulline in human blood. Consequently, ArgOX/E486Q can be adapted as an enzymatic sensor in the dehydrogenase system.

**Graphical Abstract:**

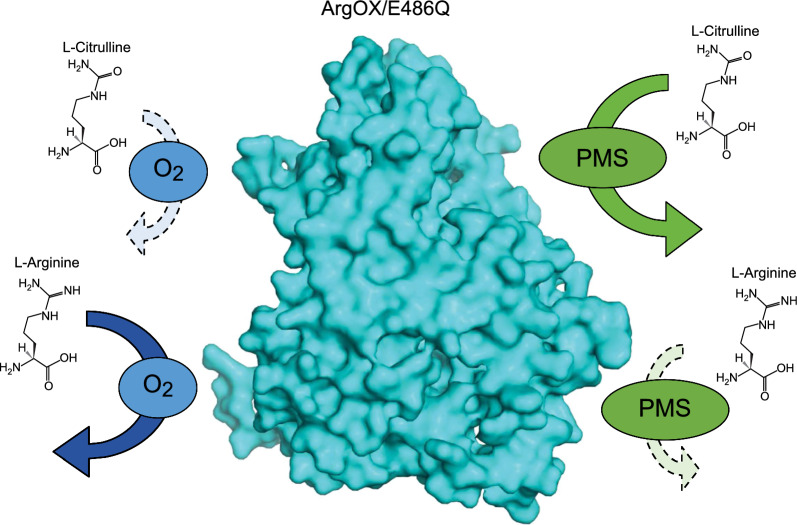

**Supplementary Information:**

The online version contains supplementary material available at 10.1186/s13568-023-01636-6.

## Key points

- ArgOX has higher substrate specificity for citrulline in the dehydrogenase assay.
- Through 3D modeling, ArgOX interacts with l-arginine via E486.
- ArgOX/E486Q can measure l-citrulline within the range of 10–500 μM.

**Supplementary Information:**

The online version contains supplementary material available at 10.1186/s13568-023-01636-6.

## Introduction

**Fig. 1:**
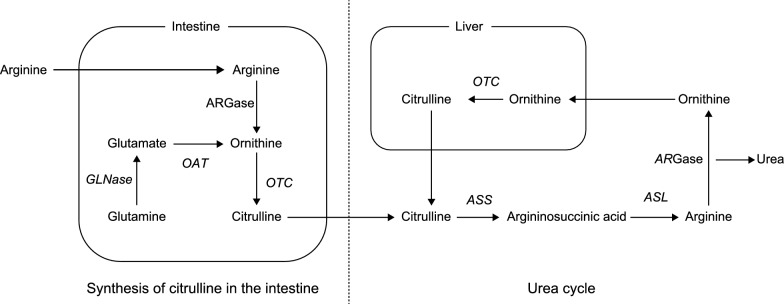
Citrulline synthesis in the intestine and the urea cycle. GLNase, glutaminase; OAT, ornithine aminotransferase; OTC, ornithine transcarbamylase; ARGase, arginase; ASS, argininosuccinate synthase; ASL, argininosuccinate lyase

Citrulline, a ubiquitous non-protein amino acid in mammals, is primarily synthesized in the small intestine by amino acid substrates, such as glutamine, proline, and l-arginine. Within enterocytes, a portion of dietary glutamine is catabolized into citrulline by several enzymes, including glutaminase, pyrroline-5-carboxylate synthase, ornithine aminotransferase (OAT), ornithine transcarbamylase (OTC), and carbamoyl phosphate synthetase-I (Aguayo et al. 2021). Biosynthesized citrulline is then metabolized through three pathways: arginine biosynthesis, the arginine-citrulline-nitric oxide cycle, and the urea cycle (Curis et al. 2005)(Fig. 1). Human blood citrulline concentrations are potential biomarkers for various diseases, including sepsis (Kao et al. 2013; Wijnands et al. 2015), OTC deficiency (Lichter-Konecki et al. 2022), and citrullinemia (Quinonez and Lee 2022), which are caused by deficiencies in OAT, OTC, and argininosuccinate synthase (ASS), respectively. Therefore, the measurement of blood citrulline concentration has applications as a diagnostic method for these diseases.

Citrulline levels are typically detected using instrumental analysis or colorimetric assays. However, instrumental methods, such as high-performance liquid chromatography (Mao et al. 2010) and liquid chromatography with tandem mass spectrometry (Jones et al. 2014), are generally expensive, have limited sample capacity, and require complex sample pretreatment. Colorimetric assays for measuring citrulline levels using diacetyl monoxime or enzymatic methods have been reported. The diacetyl monoxime method, based on the Fearon reaction, involves the formation of colored complexes between carbamide compounds, such as urea and citrulline, with diacetyl monoxime or diacetyl in an acid solution (Boyde and Rahmatullah 1980; Coulombe and Favreau 1963; Moore and Kauffman 1970). However, the high acid concentrations required render this method less practical. Enzymatic methods have been used to detect citrulline under mild, acid-free conditions by combining ASS with a pyrophosphate detection system (Kameya and Asano 2014). However, this method requires four enzymes, which can be inconvenient owing to the complexity of satisfying the conditions required for optimal enzymatic activity.

l-amino acid oxidase is an enzyme that catalyzes the direct oxidation of l-amino acids to produce 2-oxo acid, with ammonia and hydrogen peroxide (H_2_O_2_) as byproducts. Some l-amino acid oxidases exhibit broad substrate specificity for l-amino acids (Faust et al. 2007; Geueke et al. 2002). However, other amino acid oxidases, such as l-arginine oxidase (ArgOX) (EC 1.4.3.25) from *Pseudomonas sp.* TPU 7192 (Matsui et al. 2016), l-lysine oxidase from *Trichoderma viride* (Kondo et al. 2020), and l-glutamate oxidase (LGOX) from *Streptomyces sp.* X-119–6 (Yano et al. 2021) exhibit strict substrate specificity. Site-directed mutagenesis has been used to alter the substrate specificity of amino acid oxidase/dehydrogenase, with specific amino acid substitutions in LGOX leading to strict substrate specificity for l-arginine (Yano et al. 2021). However, no l-citrulline-specific amino acid oxidase has been reported. Therefore, in this study, the structural similarity between l-arginine and l-citrulline was investigated, and an l-citrulline-specific oxidase was established by introducing a mutation into an l-arginine-specific oxidase to construct a simple enzymatic detection system for l-citrulline.

## Materials and methods

### Construction of expression vectors for l-arginine oxidase mutants and transformation of *E. coli*

Based on the amino acid sequence of ArgOX from *Pseudomonas sp.* TPU 7192 (Additional file 1: Fig. S1), a sequence optimized for *Escherichia coli* codons was obtained through artificial gene synthesis(GenBank accession number LC771590). The synthesized genes were amplified using specific primers. DNA fragments were purified using a QIAquick Gel Extraction kit (QIAGEN, Venlo, Netherlands) and inserted into the *Nde*I/*Bam*HI position at the multi-cloning site of the expression vector pET-22b ( +) (EMD Millipore Sigma, Burlington, MA, USA) using an In-Fusion HD Cloning Kit (Clontech Laboratories, Inc., Mountain View, CA, USA). Expression vectors for ArgOX mutants were generated using a KOD-Plus-Mutagenesis Kit (Toyobo, Osaka, Japan) and introduced into *E. coli* BL21(DE3) (Nippon Gene, Tokyo, Japan). The primer sequences for ArgOX mutants are listed in Table 1.

**Table 1:** List of primer sequences for ArgOX mutants

Sample		Sequence	
E486H_Fw	5’-	GGTGGCTGGCATcatTGGAAAGCGAATTAT	-3’
E486N_Fw	5’-	GGTGGCTGGCATaatTGGAAAGCGAATTAT	-3’
E486Q_Fw	5’-	GGTGGCTGGCATcaaTGGAAAGCGAATTAT	-3’
E486R_Fw	5’-	GGTGGCTGGCATcgcTGGAAAGCGAATTAT	-3’
E486_Rv	5’-	AATCAGATCCAGACGATAATTCGCTTTCCA	-3’

### Determination of oxidase activity for l-arginine or l-citrulline

Oxidase activity for l-arginine and l-citrulline was assayed in a reaction mixture containing 25 mM of each substrate, 0.74 mM of 4-aminoantipyrine, 15 mM of N-Ethyl-N-(2-hydroxy-3-sulfopropyl)-3-methylaniline, 7.5 U/mL of peroxidase, 200 mM of potassium phosphate buffer (KPB) (pH 6.0), and the enzyme, in a final volume of 750 μL according to a partially modified method from a previous study (Sakaue et al. 2002). The production of H_2_O_2_ was spectrophotometrically evaluated at 37 ℃ for 3 min by measuring absorbance at 555 nm using a U-3900 spectrophotometer (Hitachi High-Tech Fielding Corporation, Tokyo, Japan). One unit of enzymatic activity was defined as the amount of enzyme that produces 1 μmol of H_2_O_2_ and 0.5 μmol of quinoneimine dye per minute at 37 ℃.

### Determination of dehydrogenase activity for l-arginine or l-citrulline

Dehydrogenase activity for l-arginine and l-citrulline was assayed in a reaction mixture containing 25 mM of each substrate, 1.8 mM of 2,6-dichlorophenolindophenol (DCIP), 15 mM of phenazine methosulfate (PMS), 200 mM of KPB (pH 6.0), and the enzyme, in a final volume of 1.5 mL at 37 ℃ according to a partially modified method from a previous study (Masakari et al. 2020). The activity was calculated by monitoring the decrease in the absorbance of DCIP at 600 nm using a U-3900 spectrophotometer. One unit of enzymatic activity was defined as the amount of enzyme that caused the reduction of 1 μmol of DCIP per minute under the assay conditions. The Michaelis constant (*K*_m_) values were determined according to the above method with 0–200 mM concentrations of l-citrulline and by fitting the results to the Michaelis–Menten equation.

### Enzyme production and purification

Recombinant BL21(DE3) *E. coli* were cultured in shake flasks containing 250 mL of ZYP-5052 medium (0.5% glycerol, 0.05% glucose, 0.2% lactose, 50 mM (NH_4_)_2_SO_4_, 50 mM KH_2_PO_4_, 50 mM Na_2_HPO_4_, and 1 mM MgSO_4_) at 30 ℃ for 24 h (Studier 2005). Cells were harvested by centrifuging at 6000 rpm at 4 ℃ for 10 min, disrupted by ultrasonication in 20 mM potassium phosphate buffer (pH 6.0), then centrifuged at 9,000 rpm for 30 min at 4 ℃. The supernatant was heated for 40 min at 60 ℃ and centrifuged at 9,000 rpm for 30 min at 4 ℃. The clear supernatant was purified via anion-exchange chromatography using a Q Sepharose Fast Flow column (Cytiva, Tokyo, Japan). The column was washed with 20 mM KPB, and the adsorbed enzyme was eluted stepwise with 20 mM KPB containing 100–500 mM NaCl. The active fraction was collected and dialyzed against 20 mM KPB (pH 7.5) using Amicon^®^ Ultra-15 Centrifugal Filters Ultracel^®^—30 K (Millipore, Billerica, MA, USA). The dialyzed enzyme solution was purified via size exclusion chromatography using a HiLoad 26/600 Superdex 200 column (Cytiva, Tokyo, Japan) equilibrated with 20 mM KPB (pH 7.5) containing 150 mM NaCl. The purified enzyme solution was dialyzed against 20 mM KPB (pH 7.5).

### Quantitation of l-citrulline using dehydrogenase assay

Quantitation of l-citrulline was assayed in a reaction mixture containing various concentrations of l- citrulline, 1.8 mM of DCIP, 15 mM of PMS, 200 mM of KPB (pH 6.0), and 2.7 μM of the enzyme, in a final volume of 1.5 mL at 37 ℃. The activity was calculated by monitoring the decrease in the absorbance of DCIP at 520 nm using a U-3900 spectrophotometer. One unit of enzymatic activity was defined as the amount of enzyme that caused the reduction of 1 μmol of DCIP per minute under the assay conditions.

### 3D model construction

A 3D structural model of ArgOX was generated via homology modeling using SWISS-MODEL [https://swissmodel.expasy.org/interactive] (Arnold et al. 2006). The crystal structure used as a template was an ancestral l-lysine oxidase K387A variant complexed with l-lysine (PDB ID:7EII). A substrate l-arginine molecule was converted from an l-lysine molecule in the resultant ArgOX homology model to avoid collision with other amino acids using the Mutagenesis Wizard of PyMOL 0.99rc6.

## Results

### ArgOX activity towards citrulline

**Fig. 2:**
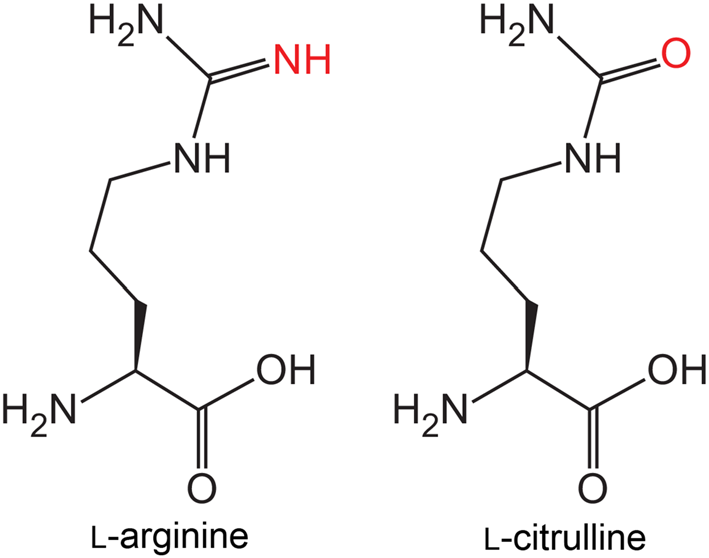
Chemical structure of l-arginine and l-citrulline

**Fig. 3:**
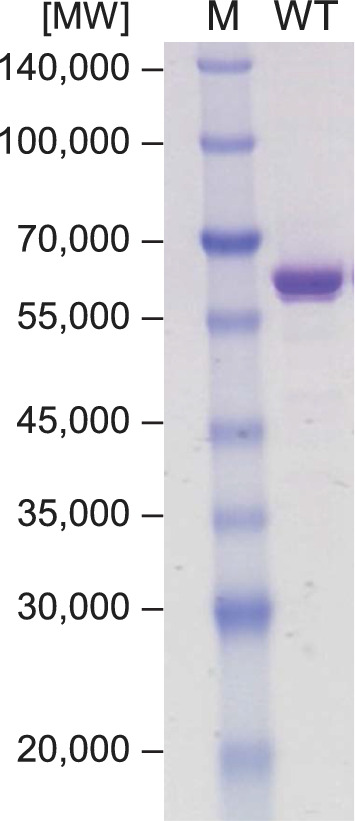
Purification of recombinant l-arginine oxidase (ArgOX). Proteins were separated on a 10–20% gradient sodium dodecyl-sulfate polyacrylamide gel electrophoresis (SDS-PAGE) gel. Lane M, protein markers with sizes shown on the left; lane WT, ArgOX (1 μg) after purification with size exclusion chromatography

ArgOX from *Pseudomonas sp.* TPU 7192 was expressed in *E. coli* to determine whether ArgOX could recognize l-citrulline, a substrate structurally similar to l-arginine (Fig. 2). The high purity of ArgOX after size exclusion chromatography was confirmed using sodium dodecyl-sulfate polyacrylamide gel electrophoresis (SDS-PAGE) (Fig. 3). The specific oxidase activity for l-arginine and l-citrulline was 89.7 ± 0.8 U/mg and 1.07 ± 0.02 U/mg, respectively. The ratio of activity against l-arginine to l-citrulline (Cit/Arg) was 1.2%, indicating that ArgOX weakly recognized citrulline as a substrate (Table 1). Certain oxidases, such as pyranose oxidase, have been reported to exhibit both oxidase and dehydrogenase activities (Herzog et al. 2019). Therefore, the activity of ArgOX dehydrogenase was evaluated. The specific dehydrogenase activity for l-arginine was 1.74 ± 0.01 U/mg, which was lower than the specific oxidase activity. The specific dehydrogenase activity for l-citrulline was slightly lower than that for the oxidase activity, and the Cit/Arg ratio was 49.5%, which was higher than that of the oxidase activity (Table 2). These results suggest that the dehydrogenase assay using ArgOX was more effective than the oxidase assay for measuring l-citrulline.

**Table 2:** Specific activity of oxidase or dehydrogenase activity for l-arginine or l-citrulline and substrate specificity

Enzyme activity assay	Specific activity (U/mg)^a^		Cit/Arg(%)
	l-arginine	l-citrulline	
Oxidase activity	89.7 ± 0.8	1.07 ± 0.02	1.20
Dehydrogenase activity	1.74 ± 0.01	0.86 ± 0.03	49.50

Values are presented as mean ± standard deviation

^a^ Specific activity was measured after incubation in 200 mM phosphate buffer (pH 6.0) with 50 mM substrate (l-arginine or l-citrulline) at 37 ℃

^b^ Cit/Arg value was calculated using the following formula:

(Specific activity of l-citrulline)/(Specific activity of l-arginine) × 100

### Construction of ArgOX mutant for enhancement of enzyme activity to citrulline

**Fig. 4:**
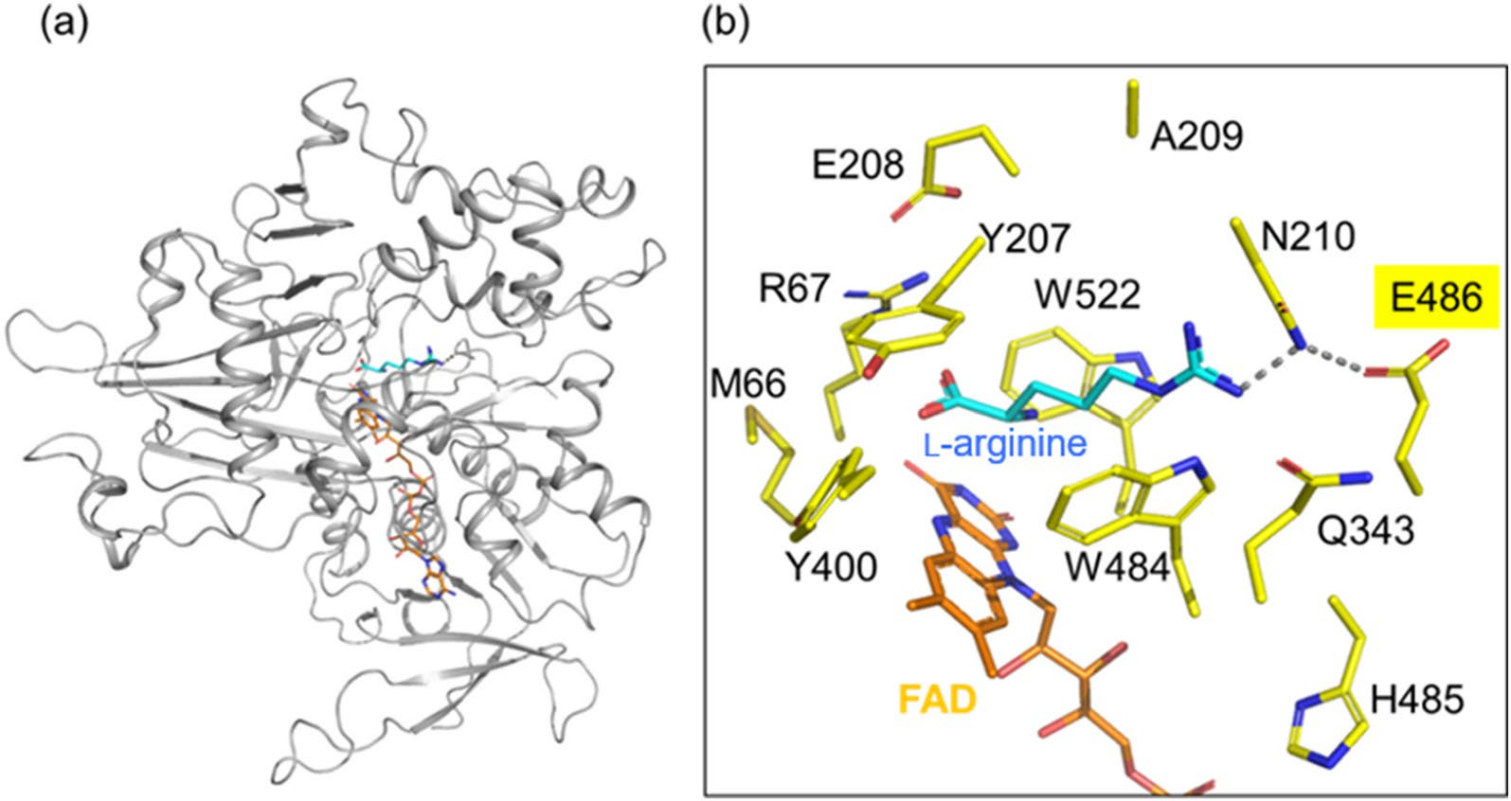
Homology modeling of ArgOX. **a** The overall model of ArgOX. **b** The ArgOX binding site for l-arginine and amino acids located within 5 Å of the substrate. Cyan: l-arginine, Orange; FAD, Yellow: amino acids

To improve ArgOX substrate specificity (Cit/Arg), we identified a target residue for site-directed mutagenesis using structure-based engineering. The 3D structural model of ArgOX was generated using SWISS-MODEL (Fig. 4), and an ancestral l-Lys oxidase K387A variant with 33.33% amino acid sequence identity served as the template (PDB ID:7EII). The ArgOX homology model was constructed from Q3 to P554 with a Global model quality estimate score of 0.59 and a QMEANDisCo global score of 0.64. The QMEAN Z-score was −3.46, suggesting that the resultant ArgOX model was of reasonable quality. At physiological pH, the guanidino group of l-arginine was protonated and positively charged, suggesting that the acidic amino acids at the active site of ArgOX interacted with the side chain of the substrate l-arginine. We hypothesized that substituting these acidic amino acids with neutral or basic amino acids would increase the l-citrulline activity and decrease the l-arginine activity. Focusing on amino acids located within 5 Å of the substrate l-arginine, we found that E486 could interact with the side chain of l-arginine (Fig. 4). Thus, site-directed mutagenesis was used to replace the glutamic acid at position 486 with glutamine, asparagine, histidine, or arginine.

### Production and enzyme characterization of ArgOX mutants

**Fig. 5:**
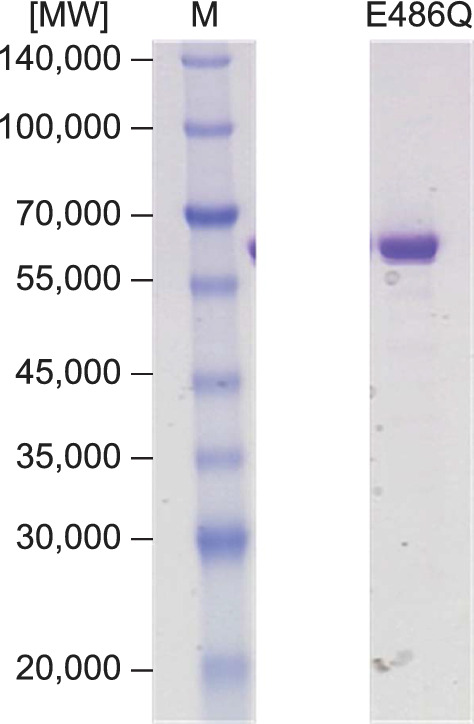
Purification of recombinant ArgOX/E486Q. Proteins were separated on a 10–20% gradient SDS-PAGE gel. Lane M, protein markers with sizes shown on the left; lane 1, ArgOX/E486Q (1 μg) after purification with size exclusion chromatography. Irrelevant sections of the gel image have been removed, and the removed areas are indicated with white lines

The activity of each mutant toward l-citrulline was investigated by expressing them in *E. coli*. SDS-PAGE revealed that the expression levels of each mutant were almost equivalent to those of ArgOX (Additional file 1: Fig. S2). The oxidase activity of ArgOX/E486N/H/R in response to l-citrulline was reduced, whereas the activity of ArgOX/E486Q was higher than that of ArgOX (Table 3). ArgOX/E486Q and ArgOX were purified to determine the Cit/Arg ratio (Fig. 5). The specific dehydrogenase activity of ArgOX/E486Q for l-citrulline was 3.25 ± 0.50 U/mg, which was 3.8-fold higher than that of ArgOX, whereas the activity for l-arginine was slightly increased compared to that of ArgOX (Table 4). The Cit/Arg ratio was 150% higher than that of ArgOX. The catalytic parameters of ArgOX and ArgOX/E486Q for l-citrulline were detected at 37 ℃ (Table 5). The *K*_m_ value of ArgOX/E486Q was lower than that of ArgOX, indicating a higher binding affinity for l-citrulline. The catalytic constant (*k*_cat_) value of ArgOX/E486Q was 2.30-fold higher than that of ArgOX. ArgOX/E486Q exhibited superior catalytic efficiency (*k*_cat_/*K*_m_) compared to ArgOX.

**Table 3:** Oxidase activity of ArgOX mutants for l-arginine and l-citrulline

Enzyme activity assay	ArgOX	Mutants			
		ArgOX/E486Q	ArgOX/E486N	ArgOX/E486H	ArgOX/E486R
l-arginine Oxidase activity (U/mL)	1.11 ± 0.01	1.73 ± 0.03	3.03 ± 0.05	1.25 ± 0.02	0.33 ± 0.01
l-citrulline Oxidase activity (U/mL)	1.06 ± 0.01	1.37 ± 0.00	0.06 ± 0.01	0.11 ± 0.01	Not detected

Values are presented as mean ± standard deviation

**Table 4:** Specific activity of ArgOX and ArgOX/E486Q for l-arginine or l-citrulline and substrate specificity

Enzyme variant	Specific activity (U/mg)^a^		Cit/Arg (%)^b^
	l-arginine	l-citrulline	
ArgOX	1.74 ± 0.01	0.86 ± 0.03	49.50
ArgOX/E486Q	2.17 ± 0.04	3.25 ± 0.50	150.00

Values are presented as mean ± standard deviation

^a^ Specific activity was measured after incubation in 200 mM phosphate buffer (pH 6.0) with 50 mM substrate (l-arginine or l-citrulline) at 37 ℃

^b^ Cit/Arg value was calculated using the following formula:

(Specific activity of l-citrulline)/(Specific activity of l-arginine) × 100

**Table 5:** Kinetic analysis of ArgOX and ArgOX/E486Q for l-citrulline

Enzyme variant	*K*_m_	*k*_cat_	*k*_cat_ /* K*_m_
	(mM)	(s^–¹^)	(s^–¹^mM^–¹^)
ArgOX	97.2 ± 3.1	2.71 ± 0.06	0.028 ± 0.001
ArgOX/E486Q	42.6 ± 2.0	6.22 ± 0.83	0.146 ± 0.013

Values are presented as mean ± standard deviation

### Quantification of l-citrulline using dehydrogenase assay

**Fig. 6:**
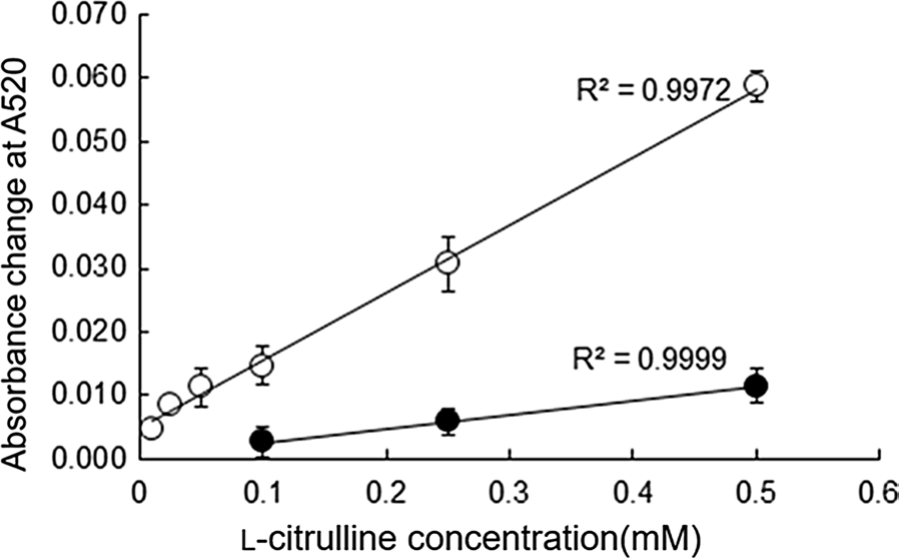
Calibration curve for l-citrulline using the dehydrogenase assay. The dehydrogenase assay was measured after incubation in 200 mM phosphate buffer (pH 6.0) with 2.7 μM enzyme (ArgOX or ArgOX/E486Q) at 37 °C (●: ArgOX, ◯:ArgOX/E486Q). Values are presented as mean ± standard deviation

The enzymatic detection of l-citrulline was performed using ArgOX and ArgOX/E486Q to evaluate the detectable concentration range of l-citrulline. Linear relationships were observed within the range of 100–500 μM and 10–500 μM l-citrulline used with wild-type and E486Q mutants, respectively (Fig. 6). ArgOX/E486Q quantified up to tenfold lower concentrations of l-citrulline than ArgOX. Given that human blood citrulline concentrations is approximately 40 μM (Crenn et al. 2008), these results suggest that ArgOX/E486Q can be used to measure l-citrulline levels in human blood.

## Discussion

In this study, a simple enzymatic method for measuring l-citrulline in a dehydrogenase assay system was developed using an ArgOX mutant with modified substrate specificity.

ArgOX exhibited a difference in the specific activity for l-arginine between the oxidase and dehydrogenase systems, with the dehydrogenase system exhibiting considerably reduced activity. Similar differences in activity between oxidase and dehydrogenase assays have been reported for other enzymes, such as pyranose oxidase and lactate oxidase; however, higher specific activities for each substrate were observed in the dehydrogenase system than in the oxidase system (Abrera et al. 2020; Noya et al. 2017). Several reports have also described the modification of specific activity in oxidase and dehydrogenase systems by introducing mutations into the enzymes (Horaguchi et al. 2014; Kim et al. 2012; Krondorfer et al. 2014). This discrepancy may be attributed to variations in the electron transfer rate from the coenzyme to the electron acceptor. For ArgOX, the rate of electron transfer to oxygen is faster than that to the electron mediator, suggesting that electron transfer to the electron mediator is impeded in the presence of oxygen, leading to lower activity in the dehydrogenase system. In future studies, it will be beneficial to measure the specific activity of dehydrogenase systems under deoxygenated conditions.

Homology modeling of ArgOX revealed that the amino acid side chain of E486 interacted with the guanidino group of l-arginine as the substrate. The ArgOX/E486Q mutant displayed high activity for l-citrulline, whereas the ArgOX/E486N/R/H mutants demonstrated reduced activity, suggesting that the glutamine at position 486 had an appropriate side chain length and a functional group that could interact with the amide group of l-citrulline by hydrogen bonding. For LGOX, the catalytic efficiency (*k*_cat_/*K*_m_) of LGOX/R305E is approximately 65-fold higher than that of LGOX/R305D. Additionally, the structure of the l-arginine complex of LGOX/R305E(PDB ID 2E1M) suggests that the side chain of D305 in LGOX/R305D is too short to interact with the guanidino group of l -arginine in the active site (Yano et al. 2021). Therefore, in modifying the substrate specificity of amino acid oxidase, it is important to select amino acids with appropriate functional groups and side chain lengths that can form ionic interactions or hydrogen bonds with the substrate.

The quantitative evaluation of l-citrulline using dehydrogenase assay showed that ArgOX/E486Q could measure with linearity within the range of 10–500 µM (Fig. 4). A previous report showed that l-citrulline could be determined with high linearity using four different enzymes within the range of 10–100 µM (Kameya and Asano 2014). The quantitative performance of the enzyme developed in this study was similar to that of previously reported enzymes used for l-citrulline detection. Regarding disease diagnosis, plasma l-citrulline concentrations > 500 µM indicate citrullinemia, a condition in which plasma citrulline levels are elevated (Quinonez and Lee 2022). However, sepsis has been reported to result in plasma citrulline concentrations of 28.8 ± 2.4 µM in the control group versus 9.9 ± 1.7 µM in septic patients (Kao et al. 2013). Both conditions fall within the range that can be measured using the enzyme developed in this study, rendering the enzyme a potentially useful tool to diagnose these diseases based on its simplicity. However, it is worth noting that the previously reported method, using the four enzymes, showed no reaction with l-arginine and displayed high specificity, whereas the method developed in this study exhibited slightly lower substrate specificity and may require further improvement.

Citrulline biosensors are yet to be commercialized. As with various amino acid dehydrogenases, it is expected that the ArgOX/E486Q could be immobilized on an electrode and used as a sensor to detect l-citrulline in food and blood (Keskin and Keskin 2014; Liang et al. 2015; Tani et al. 2009). We anticipate that the citrulline dehydrogenase developed in this study will find further applications in the future.

### Supplementary Information

**Additional file 1: Fig. S1.** Amino acid sequence of arginine oxidase from Pseudomonas sp. TPU 7192. **Fig. S2.** SDS-PAGE of crude purified enzyme from ArgOX and ArgOX mutants; proteins were separated on 10-20% SDS-PAGE gels. Lane M, protein markers of the sizes shown on the left; lane WT, E486R/H/N/Q, and pET22b in 15 μL of crude purified enzyme solution. Irrelevant sections of the gel image have been removed, and the removed areas are indicated with white lines.

## Data Availability

All data generated or analyzed during this study are included in this published article and its additional files.

## Abbreviations

ArgOX: l-arginine oxidase
OAT: Ornithine aminotransferase
OTC: Ornithine transcarbamylase
ASS: Argininosuccinate synthase
H_2_O_2_: Hydrogen peroxide
LGOX: l-glutamate oxidase
KPB: Potassium phosphate buffer
DCIP: 2,6-Dichlorophenolindophenol
PMS: Phenazine methosulfate
SDS-PAGE: Sodium dodecyl-sulfate polyacrylamide gel electrophoresis
GMQE: Global model quality estimate
*K*_m_: Michaelis constant
*k*_cat_: Catalytic constant
