# Supplementary material for: Modification of substrate specificity of l-arginine oxidase for detection of l-citrulline
Source: AMB Express. 2023 Dec 3;13:137. doi: 10.1186/s13568-023-01636-6 (PMC10694123; doi:10.1186/s13568-023-01636-6)
Supplement: Supplementary file 1 — Additional file 1: Fig. S1. Amino acid sequence of arginine oxidase from Pseudomonas sp. TPU 7192. Fig. S2. SDS-PAGE of crude purified enzyme from ArgOX and ArgOX mutants; proteins were separated on 10-20% SDS-PAGE gels. Lane M, protein markers of the sizes shown on the left; lane WT, E486R/H/N/Q, and pET22b in 15 μL of crude purified enzyme solution. Irrelevant sections of the gel image have been removed, and the removed areas are indicated with white lines. [file 13568_2023_1636_MOESM1_ESM.docx]

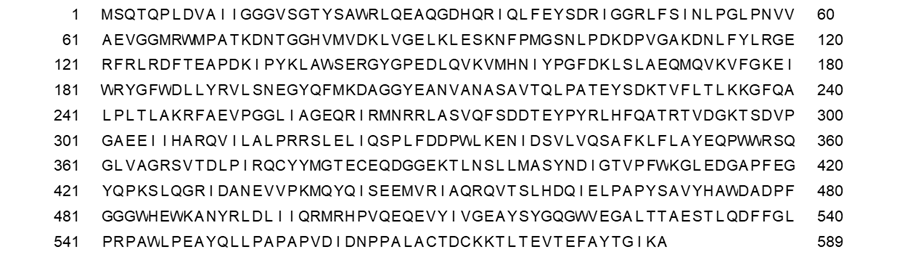


Fig. S1 Amino acid sequence of arginine oxidase from Pseudomonas sp. TPU 7192


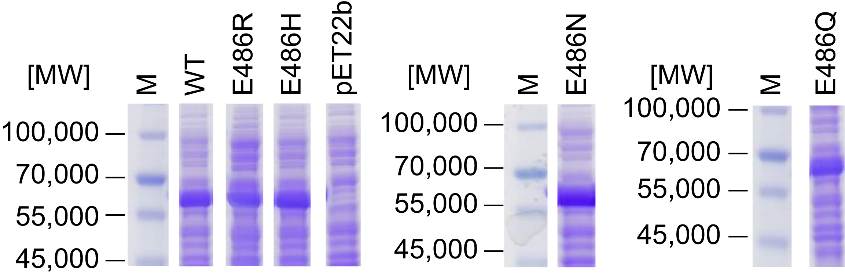


Fig. S2 SDS-PAGE of crude purified enzyme from ArgOX and ArgOX mutants; proteins were separated on 10-20% SDS-PAGE gels. Lane M, protein markers of the sizes shown on the left; lane WT, E486R/H/N/Q, and pET22b in 15 μL of crude purified enzyme solution. Irrelevant sections of the gel image have been removed, and the removed areas are indicated with white lines
